# Effect of Low Level Laser Therapy on Orthodontic Tooth Movement: A Review Article

**Published:** 2013-05

**Authors:** Soghra Yassaei, Reza Fekrazad, Neda Shahraki

**Affiliations:** 1 Department of Orthodontics, Faculty of Dentistry, Shahid Sadoughi University of Medical Sciences, Yazd, Iran; 2 Department of Periodontics, Shahid Beheshti University of Medical Sciences, Tehran, Iran; 3 Department of Orthodontics, Faculty of Dentistry, Shahid Sadoughi University of Medical Sciences, Yazd, Iran

**Keywords:** Tooth Movement; Laser; Orthodontics; Laser Therapy, Low Level

## Abstract

Increased duration of fixed orthodontic treatments leads to increased tooth root degeneration, gum inflammation and tooth caries. To decrease the time period of orthodontic treatment, it is essential to facilitate tooth movement or in other words increase the speed of bone remodeling. Use of low level laser therapy is a method for achieving this goal.

## Introduction

Orthodontic treatment uses the movement of teeth to achieve its goals that are mainly esthetic and functional. These movements result in functional forces and periodontal tissue remolding, particularly alveolar bone [1]. 

Accelerating bone remodeling may cause faster movement of the tooth with no systematic or local periodontal tissue side effects.

Long duration of fixed orthodontic treatment, which usually lasts for 2-3 years, is accompanied by side effects such as root resorption, gingival inflammation and dental caries [2]. Researchers have tried various methods to increase orthodontic tooth movement (OTM) including surgical cortical incisions around the teeth according to regional acceleratory phenomenon [3-4], injection of chemical substances such as local prostaglandins [5-6], vitamin D3 [7-8] and osteocalcin [9] around the sockets that can lead to acceleration of tooth movement by altering bone modeling and remodeling [10-11]. Distraction osteogenesis also leads to acceleration of tooth movement by formation of callus [12-13], Furthermore, vibration [14], electromagnetic fields [15] and electric current [16] are other methods used to increase OTM.Though most of these methods have been successful, they have risks and untoward effects. For example, injection of prostaglandin E2 is very painful and can cause root resorption [17] and repeated injections of vitamin D3 and/or parathyroid hormone may have undesirable effects. Recent studies have shown that low level laser therapy can be used for acceleration of tooth movement and alveolar bone remodeling. They are non invasive, easy to use, cheap and do not need any special expensive machinery [18-21].

The pressure-tension theory of orthodontic tooth movement is the most accepted model for explaining how teeth move through the bone. It states that when a sustained force is applied to a particular point on a tooth surface, areas of the tooth’s root are pushed against the alveolar bone creating compression in the PDL space, and other side that are pulled resulting in tension in the PDL complex. Areas of pressure and tension within the PDL create morphologic and functional changes in the cells of the PDL. Histological samples of compressed PDL show less organization and general disruption of the structural elements, including compressed blood vessels [22]. 

This compression leads to a subsequent decrease in the availability of oxygen and other key cellular factors that are responsible for PDL maintenance. Areas of tension do not show structural disorganization under the microscope. With limited stretching of the PDL fiber bundles, an increase in blood perfusion is observed and levels of oxygen availability will decrease [23]. 

The changes in the microenvironment of the PDL initiate chemical signals to restore equilibrium. These chemical messengers elicit a chain of biochemical events leading to the activation of osteoclasts and osteoblasts. 

These are the principle cells responsible for osseous modeling and remodeling that occur as teeth move from one position to the next within bone [24]. The pressure tension theory also accounts for various types of bone resorption seen in histological sections. It is described by different responses of the tissues to the amount of force used to compress the PDL. 

Heavy forces lead to undermining resorption of the bone. High forces placed on teeth compress PDL components tightly and the blood vessels are occluded [25]. Without a blood supply, cells in the environment die due to the lack of oxygen and nutrients. This results in sterile necrosis in the PDL, a process also referred to as hyalinization [26]. Macrophages and foreign body giant cells remove remnants of the dead PDL while osteoclasts begin resorbing lamellar bone from the adjacent alveolar bone marrow cavities. This resorption from underside of the bone next to the marrow spaces continues until all lamina dura is removed and the soft tissues of the hyalized area are reached. When all the necrotic tissue is removed by the phagocytic cells, tooth movement occurs [27]. 

Light forces lead to frontal resorption of the bone. Light forces transmitted to the teeth will compress the PDL tissue, but total occlusion of the microvasculature will not occur. Within minutes of the initial force, the blood flow decreases and the change in the microenvironment results in the release of chemical messengers. They cause metabolic changes in the tissue within a few hours. Cellular differentiation occurs in preosteoclast cell lines and bone resorption is initiated adjacent to the PDL space. The bone resorbed in this manner is called frontal resorption and occurs in a more continuous manner allowing sustained movement of the teeth [28].

**Laser definition and characteristics:**

The acronym laser stands for “light amplification by stimulated emission of radiation” [29]. Lasers, like all light, behave as a particle and a wave, but also possess several unique characteristics. Lasers are monochromatic, or contain light of a very narrow bandwidth. Monochromatic light wavelengths are well ordered and remain synchronized with one another. This quality, described as coherence, means that all of the laser waves are the same shape and have the same frequency and phase [30]. 

Laser can be delivered in continuous or interrupted emission modes. As suggested by its name, a laser operated in continuous mode delivers a continual stream of laser light. Interrupted mode can be divided into gated pulsed and free running pulsed mode. Gated pulsed mode was initially achieved when a mechanical shutter momentarily blocked the transmission of the laser light that otherwise would be functioning in continuous mode. It can also occur by turning the laser on and off. 

Superpulsed mode, a form of gated pulsed mode, dramatically shortens the pulse width to less than 50 milliseconds. This allows for a simultaneous increase in the laser’s photons’ peak power of about 10 times that of continuous wave power measurements [31]. 

Dentistry lasers can be classified into high power and low level laser therapy. High power lasers have an output power of 1 watt and are used for cutting soft and hard tissue. Their energy density ranges from several 100 watts to several thousand watts per square centimeter. In orthodontics, LLLT [low level laser therapy] is used for reduction of pain [32-34] and increases bone absorption in the length of mid palatal sutures during expansion [35]. Most of the primary studies regarding low level laser therapy have been with He-Ne lasers with 632.8 nm wavelength. This laser was the first commercial laser that has been used extensively [36].

 The penetration of LLLT in a tissue is related to certain factors. The most important absorption coefficient is the substance on which the laser is shined upon.

Baxter and Diamantopoulos [37] stated that laser wave length and energy density are the most important factors determining the tissue response. 

Mester et al. stated that energy density in the 0.5-4 J/cm2 is the most effective range in start of a photobiological tissue reaction [38]. 

Van Gemert and Welch stated that the maximum penetration of infrared lasers in bloodless tissues is 1 cm [31]. Laser manufacturing companies state that the penetration of low level laser therapy is more than 5 cm [39]. Anyway, only a fraction of the primary laser reaches this depth. One study has shown that 850 nm laser with an output power of 100 mw loses 66% of its power after 1 mm penetration [40]. 

In order to reach the photo reactive parameters of low level laser therapy, factors including light intensity, power output, power density, total irradiation and energy density are important. Van Breugel et al. reported that power density is more important than the total dose in start of biomodulation [41]. 

Sommer et al. believe that energy density and light intensity are more important biomodulation factors [42]. Kujawa et al. reported that increase in acetyl cholinesterase and internal protein storage following LLLT results in increased cellular longevity [43-44].

## MATERIALS AND METHODS

Search engine:

We have used full text articles in Pubmed, Highwire and Google Scholar with search title “orthodontic tooth movement and LLLT” in English language. Studies in the parameters of laser including wave length and average power or laser of energy.

## RESULT

**Laser and orthodontic tooth movement:**

Studies that evaluated the effect of low level laser therapy on the rate of tooth movement are demonstrated in Table 1. 

The primary study in this field was by Saito et al [35] in 1997 Regeneration of mid-palatal suture in mice has been studied using low level laser therapy (GaAlAS with an output of 100 mw for 3-10 minutes per day for 7 days). The results showed that lasers can increase the speed of regeneration of bone in the midpalatal suture and the rate is related to dose, time and frequency of the rays.

**Mechanisms of laser effects on orthodontic tooth movement rate:**

1-During application of orthodontic forces on the teeth, osteoclasts are increased on the compressed side and osteoblasts on the traction side. The increased number of cells stimulates bone remodeling around root and leads to tooth movement [45-48]. 

Resorption and deposition occur equally in physiologic remodeling and the bone mass does not change. Intact skeletal integrity of the body is the result of dynamic balance between osteoblasts and osteoclasts [49]. The rate of remodeling is basically associated with osteoblasts because osteoblasts are responsible for induction of proliferation and differentiation of the osteoclastic cells. These factors include tumor necrosis factor (TNF) and receptor activator of nuclear KB ligand (RANKL) [50]. RANKL bonds to its receptor which is receptor activator of nuclear KB (RANK) and induces osteoclastogenesis and activates osteoclasts [49-51].

On the other hand, osteoprotegrin is a cytokine that is produced by osteoblastic and bone marrow cells and inhibits osteoclastogenesis. Because it is able to bond to RANK and prevents bonding of RANKL, it leads to inhibition of osteoclastogenesis. RANKL and osteoprotegrin therefore regulate bone resorption. RANKL levels are increased in PDL and gingival sulcus fluid during OTM, while osteoprotegrin decreases significantly. Low level laser therapy cause RANKL increase in periodontal ligament and it can increase the rate of tooth movement during orthodontic treatment [51]. Aihara et al. evaluated preosteoclast-like cells to measure the amount of RANK after radiation in vitro. Immunohistological staining and RT- PCR expressed higher levels of RANK and RANKL in the laser therapy group as compared to the control group [52].

**Table 1:** Studies That Evaluated the Effect of Low Level Laser Therapy on Tooth Movement Rate

**Author**	**Study Design**	**Mode of Laser**	**Wave Length of Laser**	**Effect on OTM or Bone**
**Saito (35)**	Animal study	GaAlAs 100mw		+
**Kawasaki (18)**	Animal study	GaAlAs 100-600mw	830nm	+
**Cruz (19)**	Human study (random split mouth design)	GaAlAs	780nm	+
**Limpanichkul (2)**	Human study (random split mouth design)	GaAlAs 100mw	850nm	-
**Goulart (45)**	Animal study (split mouth double blind design)	GaAlAs 70mw	780nm	+
**Seifi (20)**	Animal study (blind random ization methods)	KLO3 & Optodan	850nm&630nm	_
**Yamaguchi (46)**	Animal study	GaAlAs 100mw	810nm	+
**Youssef (32)**	Human study (randomized clinical trial)	GaAlAs 100mw	809nm	+
**Fujita (21)**	Animal study	GaAlAs 100mw	810nm	+
**Yoshida (47)**	Animal study	GaAlAs 100mw	810nm	+
**Marquezan (48)**	Animal study	GaAlAs 100mw	830nm	_

Kim et al. [53] also evaluated the amount of RANK/ RANKL using 2 immunohistochemistry analyses. They realized that RANKL exists in both groups of laser therapy and control from the beginning of the study, but RANKL levels were higher in the laser group from the beginning to the end of the study. 

2- Type I collagen fibers exist in high levels in PDL space and increased fiber turnover is necessary for tooth movement. In addition, fibronectine which is spreaded all over in the mesenchyme of PDL supports proliferation and differentiation of the fibrolasts and production of type I collagen and fibronectin increases in response to mechanical stresse or in other words during OTM [54].

Based on a study conducted by Kim et al. [53], application of low level laser therapy of GaAlAs with a wave length of 808 nm and an output power of 96 mw causes increase of fibronectin and type I collagen levels from the first day and it remains significant to the end of the experiment in laser and control groups. Kim et al. [53] finally concluded that low level laser therapy facilitates the turnover of connective tissue during tooth movement. Ozawa et al. [55] studied various aspects of LLLT in vitro. They irradiated osteoblast-like cell cultures at various stages of differentiation and observed bone nodule formation. They showed that bone nodule formation occurred more in the rat calvarial cells when treated with early irradiations, but this was not the case with later treatments. 

Nakyamada et al. and Nishiguchi et al. declared fibronectin can induce up-regulation of RANKL which leads to osteoclastic differentiation and so fibronectin has a great role in bone and PDL turnover [56-57].

3- Low level laser therapy increases osteoblastic cell proliferation and can therefore stimulate osteogenesis and increase bone density on the traction side [55].

4- Macrophage-colony stimulating factor (M- CSF) not only stimulates proliferation of osteoclastic progenitors, but it also affects their differentiation into mature osteoclasts. Based on a study carried out by Yamaguchi et al., low level laser therapy can increase M- CSF on the compressed side and may also increase osteoclastogenesis leading to tooth movement [46]. Cell response to low level laser therapy stimulation is induced by photoreceptors of mitochondrial breathing chain [58]. In general, the effect of low level laser therapy on cells depends on wave length and molecular absorption of laser beam is required for any kind of laser effects [59-61]. Vascularization plays a key role in OTM because both frontal and undermine resorptions require blood vessel supply. Garavello et al. [62] drilled a hole in the rat's tibia and applied low power intra cutaneous laser for 14 days. Histologic samples showed increased deposition of osseous matrix 7 days after radiation in the laser group compared to the control. Other investigations are also representative of the increased vascularity after laser therapy in the non-osseous tissue [63-65] and also increased molecular factors related to vascular proliferation [66-67].

## Discussion

Most of the 11 studies that evaluated the effect of low level laser therapy on the rate of tooth movement concluded that low level laser therapy causes increase in tooth movement [19,21,32,47]. A few studies concluded that low level laser therapy has no effect on orthodontic tooth movement [OTM] due to the high amount of energy or wave length of laser because the amount of energy that each tissue receives can affect the way it responds [2,20,48]. Lasers with wave lengths of 860 nm and 850nm were applied in experiments of Limpanichkul et al.[2] and Seifi et al.[20], but other studies used lower wave length lasers [780, 800 and 810 nm]. This could be a reason for different amounts of tooth movement. 

In Seifi's experiment, visible wave lengths such as 630 nm did not result in an increased rate of tooth movement because the penetration depth of infrared is higher than visible waves and laser must stimulate bone and PDL cells [20].

## CONCLUSION

Based on different researches, it may be concluded that low level laser therapy may increase the rate of tooth movement during orthodontic treatment by the following mechanisms:

- Increasing levels of RANKL in PDL which leads to increased osteoclastogenesis.

- Increased M- CSF levels and osteoclastogenesis.

More researches are needed to determine the effect of low level laser therapy on tooth movement with special attention to laser parameters. 
